# Energy Landscapes and Structural Plasticity of Intrinsically Disordered Histones

**DOI:** 10.1021/acs.jcim.4c02269

**Published:** 2025-08-06

**Authors:** Rafael G. Viegas, Hao Wu, Murilo N. Sanches, Garegin A. Papoian, Vitor B.P. Leite

**Affiliations:** † 119519 Federal Institute of Education, Science and Technology of São Paulo (IFSP), Catanduva, SP 15.808−305, Brazil; ‡ Department of Physics, Institute of Biosciences, Humanities and Exact Sciences, 135131 São Paulo State University (UNESP), São José do Rio Preto, SP 15054-000, Brazil; § Oncology Chemistry, 12295 AstraZeneca, 35 Gatehouse Dr, Waltham, Massachusetts 02451, United States; ∥ Biophysics Program, Institute for Physical Science and Technology, University of Maryland, College Park, Maryland 20742, United States; ⊥ Department of Chemistry and Biochemistry, University of Maryland, College Park, Maryland 20742, United States

## Abstract

Intrinsically disordered proteins (IDPs) are characterized by their lack of a stable 3D structure, enabling them to adopt multiple conformations and participate in various cellular processes. This study investigates the conformational dynamics of histone tails, specifically the H4 tail and the linker histone H1, focusing on the effects of post-translational modifications (PTMs) such as acetylation. Utilizing the energy landscape visualization method (ELViM), we projected the conformational space of wild type and acetylated forms of the H4 tail, revealing significant insights into their structural heterogeneity and preferential ensembles. This approach demonstrated that acetylation reduces the conformational heterogeneity of the H4 tail and introduces regions within the conformational space uniquely occupied by each form, which may correlate with specific biological functions. Furthermore, the conformational space of the linker histone H1 was analyzed, illustrating how its structural heterogeneity is influenced by nucleosome binding modes. This work highlights the critical role of conformational plasticity and PTMs in regulating the multifunctionality of IDPs, thereby enhancing our understanding of their contributions to chromatin dynamics and cellular regulation.

## Graphical abstract

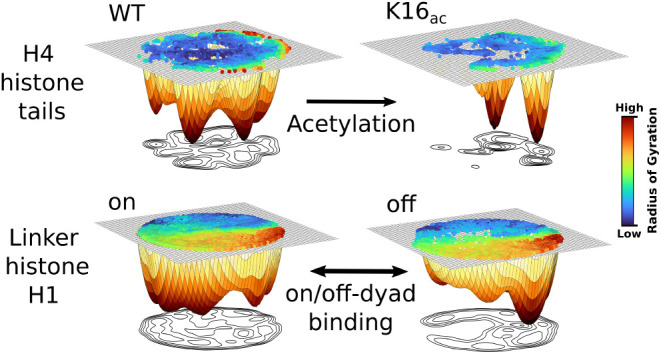

## Introduction

In recent decades, our understanding of protein function has been significantly transformed by the discovery that numerous proteins remain functional despite lacking a well-defined structure.^1–3^ These proteins, known as intrinsically disordered proteins (IDPs), have been recognized as central hubs in cellular protein interaction networks^4,5^ and play crucial roles in various cellular phenomena such as cell signaling and regulation^6–8^ and in the development of severe pathological conditions.^9–12^

Owing to its inherent flexibility, an IDP is better characterized as a functional heterogeneous ensemble, in which the protein can access multiple thermodynamically stable states separated by low energy barriers.^12,13^ This flexibility is crucial for the multifunctionality of IDPs, as different preferential ensembles can be related to distinct biological functions.^14–16^ These functions can be switched “on” or “off” through post-translational modifications (PTMs) such as acetylation, which promote local changes in physicochemical properties like charge and flexibility.^17,18^ These modifications reshape the energy landscape and the preferential ensembles of IDPs, thereby regulating their activity. In addition, the conformational flexibility can lead to what is termed conformational noise, which may affect cellular fate by introducing variability in signaling and regulatory processes.^5,19,20^ Additionally, this same plasticity can lead IDPs to engage in promiscuous interactions, which are often linked to pathological states.^12^

Histone tails, the unstructured N-terminal regions protruding from the nucleosome, exemplify the regulated multifunctionality of IDPs through PTMs. In this study, we focus on the H4 tail and its acetylated forms (Figure 1). The H4 tail mediates both intra- and internucleosome interactions, playing a critical role in regulating chromatin structure.^21–25^ Despite its high flexibility, theoretical studies suggest that the energy landscape of histone tails is not entirely disordered but reveals structural organization. Potoyan and Papoian, using all-atom REMD simulations, demonstrated that the H4 tail deviates from random coil behavior, adopting a multibasin energy landscape enriched in beta-hairpin conformations.^26^ Röder et al.^27^ using the path sampling technique^28^ also characterized the H4 tail as a multifunnel system comprising five distinct minima, separated by high-energy barriers. In this context, the dynamics of such a multifunctional system likely involve frequent transitions between basins, with certain stable, long-lived states potentially corresponding to distinct biological functions.^26,27^

The effects of acetylation on the H4 tail has also been studied, revealing that PTMs can significantly impact its function and the overall chromatin structure.^13,25,27,29–32^ Acetylation of the H4 tail has been shown to modify the free energy landscape of chromatin, weakening internucleosome interactions and disrupting the organization of the chromatin fiber.^25,33^ Specific lysine acetylations have also been well-characterized; for instance, acetylation of K16 dramatically impacts the conformational heterogeneity of the H4 tail, reducing its flexibility and giving rise to unique structures not observed in the wild-type form.^30–32^

In this study, we applied ELViM,^34,35^ a multidimensional projection technique to perform a differential analysis of conformational ensembles of the histone H4 tail and its acetylated forms. Since ELViM’s metric is based solely on the internal coordinates of the α-carbons, it allows for estimating structural similarity between all conformations, resulting in a single effective conformational space simultaneously occupied by all forms of H4 tails. This approach has previously been used to analyze both molecular dynamics trajectories of IDPs^36^ and ensembles generated by integrative approaches.^37^ The unified conformational space provides an intuitive visualization of the dominant conformational states, enabling a direct comparison of how acetylation reshapes the energy landscape, affecting the preferential conformations and their structural heterogeneity. Furthermore, ELViM’s projection effectively distinguishes regions of the conformational space uniquely populated by individual H4 variants.

Another key histone is linker histone H1, which binds to DNA between nucleosomes (Figure 1). H1 is a lysine-rich, highly positively charged protein that plays a crucial role in chromatin compaction by stabilizing higher-order structures.^13,38^ In eukaryotes, H1 consists of a short N-terminal domain (NTD), a central globular domain (GD), and a long, highly disordered C-terminal domain (CTD).^39^ The disordered CTD is vital for high-affinity binding and promotes the folding of nucleosome arrays.^13,40–42^ H1 can bind to the nucleosome in two distinct modes: “on-dyad” and “off-dyad,” where the dyad axis refers to the pseudo-2-fold symmetry of the nucleosome.^43,44^ These different binding modes can shift during chromatin folding, leading to various levels of chromatin condensation.^13,45^

Additionally, we also analyzed the conformational landscape of the linker histone H1, as described in the reference.^42^ In this coarse-grain model, full-length H1 was simulated in the presence of a nucleosome particle. The effective conformational space for H1 was projected, and preferential conformational states were described. In addition, we examined how these preferential conformations relate to the nucleosome binding mode.

As a conclusion, ELViM projections show that the accessible regions of the single effective conformational space of the analyzed histones are shifted by either acetylation, for H4 tails, or binding mode, for linker H1. This modulation of their conformational space likely regulates their distinct biological functions.

**Figure 1:**
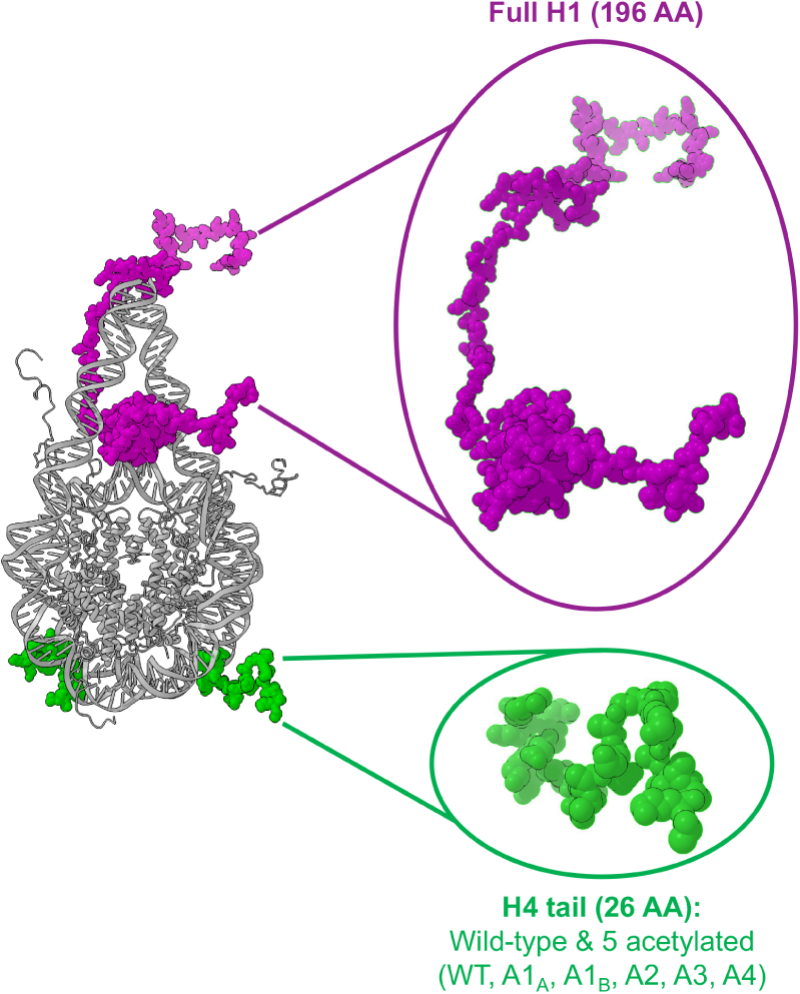
Cartoon structures of two disordered histone domains, H4 tail and H1, are shown along with the entire nucleosome particle. The wild type and five acetylated models of the H4 tail were investigated in this study. The full-length H1, including the disordered N-ter and C-ter and the globular central domain, was simulated with a nucleosome particle. See Simulation Details for further description of the simulated systems and setup.

## Methods

### Atomistic Simulations of the H4 Tail

Atomistic MD simulations of all the H4 tail systems studied in this work were performed in a previous publication (Winogradoff et al.^31^). The simulation trajectories were directly adopted in this work for analyses with the permission of the corresponding author. The simulated H4 tail systems include the wild type (WT) and five post-translational modified ones with different levels and sites of lysine acetylations (A1_A_, A1_B_, A2, A3, and A4, named as H4–K16_ac_, H4–K5_ac_, H4–K8_ac_K16_ac_, H4–K8_ac_K12_ac_K16_ac_, and H4–K5_ac_K8_ac_K12_ac_K16_ac_ in Winogradoff et al.^31^). The initial conformation of the WT H4 tail was obtained from a previous work,^26^ based on which all the acetylated models were prepared using *xleap* tool in AmberTool12. All the H4 tail simulations used amber99SB*^46^ for proteins, ions94 ^47^ for ions, and TIP3P^48^ for water as force fields, with the NVT ensemble, 2 fs time-step, and Langevin thermostat. Replica exchange molecular dynamics (REMD)^49^ simulations were performed to enhance sampling of the disordered H4 tails, resulting in ∼60 replicas at different temperatures, totaling 6 μs of simulated trajectories for each system. The final 90 ns trajectories of the replica at 300 K were used for further analyses. These trajectories were originally published by Winogradoff et al.,^31^ where simulation protocols and convergence analyses – such as radius of gyration and secondary structure probability distributions – are provided in detail in the Supporting Information.

### Coarse-Grained Simulations of H1-Nucleosome

The H1-nucleosome trajectories were first published by Wu et al.,^42^ and in this present work, we reanalyzed them with permission of the corresponding author. In the previous publication, Wu et al.^42^ modified a CG force field called AWSEM,^50^ a protein model with both physics-based and bioinformatics-inspired potential and implicit solvent, to simulate the full-length H1, including the disordered N- and C-terminus, bound to the entire nucleosomal particle. This hybrid force field “AWSEM-DNA” incorporates AWSEM,^50^ its intrinsically disordered protein branch AWSEM-IDP^51^ and a CG DNA force field 3SPN.2 ^52^ to model protein–DNA complex, with high accuracy and sufficient sampling efficiency, especially for a large system with disordered domains. The initial conformation of the H1-nucleosome was obtained from an experimental structure (PDB: 5NL0),^53^ with N- and C-terminus modeled using MODELER.^54^ A total of 50 replicas were simulated using a 5 fs time step, NVT ensemble, and Langevin thermostat, totaling 3 μs of production trajectories (in CG time scale, approximately corresponding to milliseconds of atomistic simulation time). Coordinates of H1’s C_α_ atoms were extracted for analyses in this study. For a detailed description of the CG force field and additional simulation details, see Wu et al.^42^ and its Supporting Information.

### Energy Landscape Visualization Method (ELViM)

ELViM initially assigns a dissimilarity, which is based on internal distances, to all pairs of conformations. The dissimilarity metric comes from the order parameter *Q*_*w*_, which has been widely used to describe protein folding and dynamics.^55^ When evaluated pairwise, *Q*_*w*_ is a measure of structural similarity between the conformations (*k*, *l*), and it is given by

1 $$Q_{w}^{k,l}=\frac{1}{N_{p}}\underset{i,j\in \mathrm{pairs}}{\sum }\exp\left[\frac{-{\left(r_{i,j}^{k}-r_{i,j}^{l}\right)}^{2}}{2\sigma_{i,j}^{2}}\right]$$

where $r_{ij}^{k}$ $\left(r_{ij}^{l}\right)$ is the Euclidean distance between *C*_α_ atoms (*i*, *j*) from conformation *k*(*l*), and *N*_*p*_ is the total number of *C*_α_ pairs. σ_*i*,*j*_ is a weighting parameter that depends on the sequence distance between *C*_α_ pairs. It is given, in angstroms, by σ_*i*,*j*_ = |*i* – *j*|^0.15^.^55^ Finally, the pairwise dissimilarity is $\delta_{k,l}=1-Q_{w}^{k,l}$ . In this way, δ_*k*,*l*_ is unitless, and it equals zero when structures are identical and approaches one when structures are very different. This dissimilarity metric is applied to all pairs of conformations, resulting in a dissimilarity matrix. Then, using the force-scheme technique,^56^ ELViM performs a dimensionality reduction so that protein conformations are represented on the plane by points whose proximity correlates with their dissimilarity. Specific regions of the conformational space are characterized by extracting representative conformations defined as local conformational Signatures (LCSs).^34^ An LCS is obtained from manually selected arbitrary regions by calculating a matrix of distance-RMSD values and finding the conformation that minimizes the average distance-RMSD for the selected group. The LCS displays this conformation superimposed on its *n* closest neighbors according to the distance-RMSD values.

In this work, we projected the effective conformational space for different systems comprising histone models: the H4 histone tail and linker histone H1. From each H4 trajectory, a conformation was selected every 14 frames after discarding the first 4000 frames for further equilibration. In this way, 6000 conformations from each H4 model were selected, resulting in a single conformational space with 36000 conformations. For the second system, the full-length H1 model, the coordinates of the H1 *C*_α_ atoms were extracted, and conformations were selected every 13 frames after discarding the first 4000 frames, totaling 30005 conformations.

## Results and Discussion

### Exploring the Effect of Acetylation on H4 Histone Tails’ Conformational Landscape

To generate a visualization of the effects of acetylation on the conformational space of the H4 histone tails, we applied ELViM to project a single effective conformational space populated by the wild type and the acetylated (A1_A_, A1_B_, A2, A3, and A4) models. The resulting space is shown in Figure 2A, in which each dot corresponds to a sampled conformation. In an ELViM projection, the Euclidean distance between points represents structural similarity, with closer points corresponding to more similar conformations. Unlike clustering approaches that divide conformations into distinct groups, ELViM maps them onto a continuous conformational landscape, as shown with the radius of gyration (*R*_g_) for each conformation through the colormap. The *R*_g_ values vary smoothly over the projected space, with lower values in the inner-left region and higher values populating the outer-right region of the projection. It is worth noting that point distribution is not uniform over the projected space, since the projection’s minimization procedure tends to group similar structures, and these groups of similar structures are placed apart based on their mean structural dissimilarity. As an example of ELViM’s capability to group similar structures, Figure 2B presents representative conformations as LCSs (see Methods), each one consisting of 30 superposed structures.

**Figure 2:**
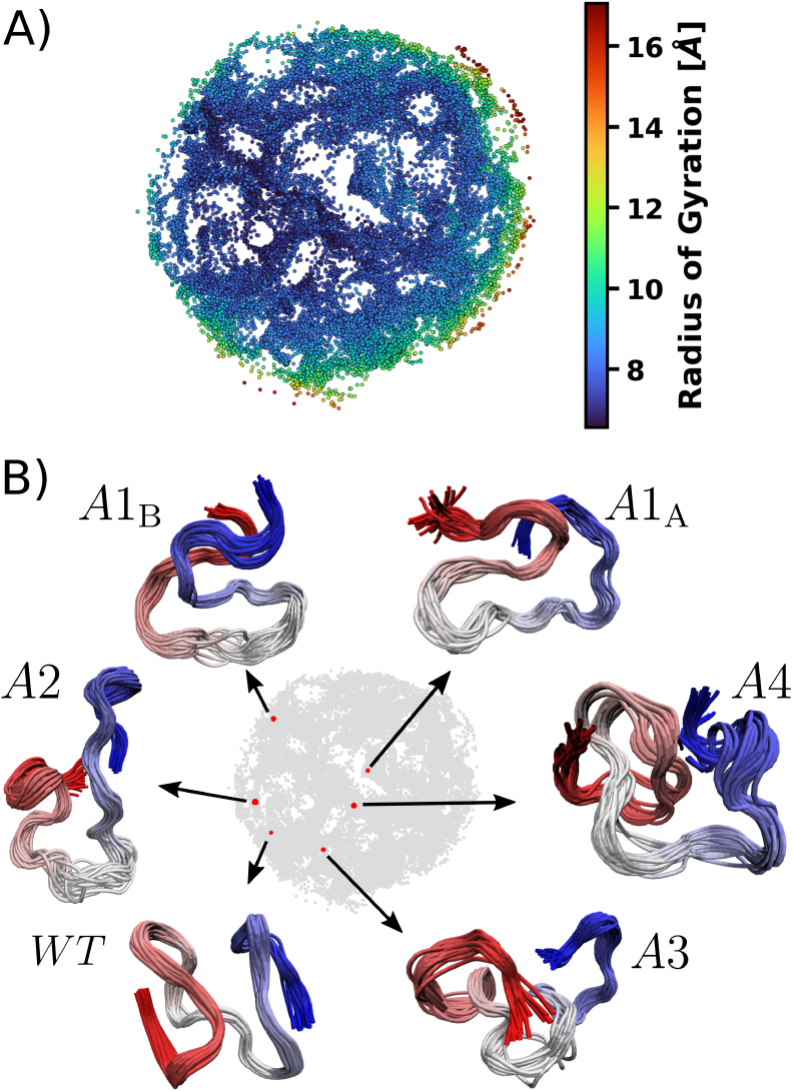
Effective conformational space for the H4 tails. (A) Each sampled conformation is represented by a point in the projected space. Points are colored based on *R*_g_ values. (B) Local conformational signature (LCS). To illustrate the single effective conformational space generated by ELViM, an LCS containing 30 aligned conformations is shown for each H4 tail system. The points colored in red highlight their location in the projection, while the backbones are depicted with N-terminal residues drawn in red.

Once the global conformational space is projected, it is possible to explore the conformational heterogeneity of each H4 model across the projected space. The conformational space for each model is shown in Figure 3. In this figure, points from each labeled model are colored according to their *R*_g_ values, while conformations from other models are shown in gray. It is possible to note that individual spaces are not uniformly distributed, with some models having access only to some regions of the entire space. The WT and A2 models have the most homogeneous landscape, spread widely across the projection. In contrast, it is clear that the conformational space of monoacetylated A1_A_ is greatly reduced. The A3 and A4 models also tend to concentrate conformations in specific regions of the conformational space. This result is in agreement with a previous analysis showing that cumulative acetylation reduces the conformational heterogeneity of the system.^31^ In addition, Figure 3 reveals that some regions of the entire space are populated by a single model. The most important one is encircled by a red line and is uniquely populated by the A1_A_ model. These uniquely populated regions indicate that acetylation not only affects the H4 conformational heterogeneity but also reshapes the H4 energy landscape, leading to different subsets of dominant structures.

**Figure 3:**
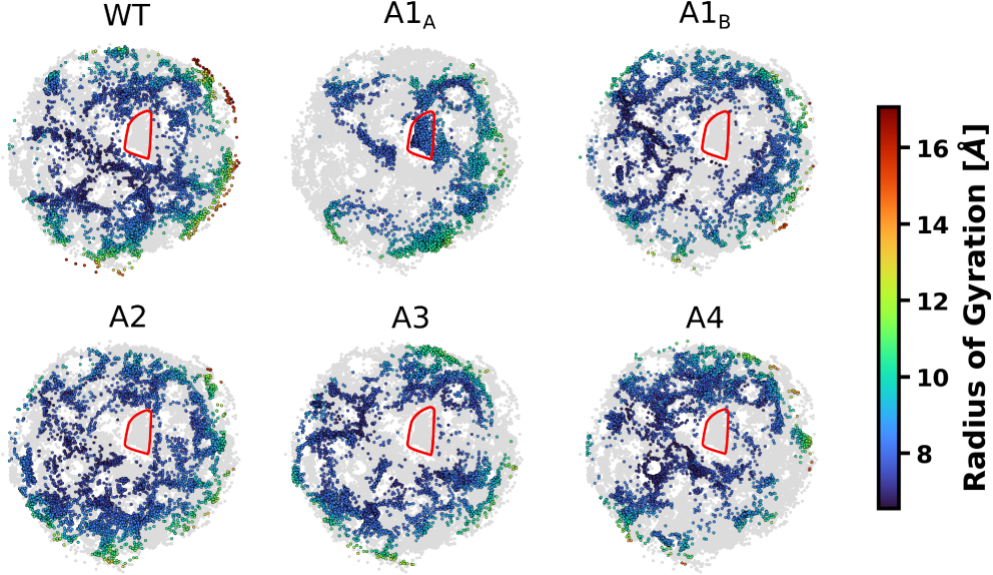
Effective conformational space of each H4 model. Points from each labeled model are colored based on their *R*_g_ values. Points from other models are shown in gray. In addition to the change in conformational heterogeneity, the modified models can sample exclusive regions of the conformational space. The most important example is the A1_A_ model, which has an exclusive region encircled by a red line.

To provide a more detailed structural characterization, we have replotted the effective conformational space for each H4 model in the Supporting Information, with data points colored based on local density. The density was calculated using a Gaussian kernel density estimate (KDE) and reflects the effective density of states generated by ELViM. For each model, six LCSs were extracted from high-density regions, providing representative structures for each model (Figures S1–S6).

Additionally, contact maps for each LCS are included. Figure S7 shows how the relative solvent accessible surface area (SASA) of the basic patch (residues K16R17H18R19K20) varies across the effective conformational space. The basic patch is a positively charged region that interacts with acidic patches on both DNA and H2A/H2B.^16,23,57^ It has also been acknowledged that acetylation increases the helical content of histone tails.^29,30,58^ In line with this, Figure S8 presents the helical fraction of each conformation in the ELViM projection. These results illustrate how a single effective conformational space can be leveraged to examine the accessibility of different biophysical properties across distinct regions.

In order to highlight regions of the entire conformational space that are uniquely populated by each H4 tail model, we calculated the local relative fraction of each model. We adopted a 27 × 27 grid and defined the relative fraction, $F_{i,j}^{w}$ , of system *w* in the grid bin (*i*, *j*) as

2 $$F_{i,j}^{w}=n_{i,j}^{w}/N_{i,j}$$

where $n_{i,j}^{w}$ is the number of conformations from model *w,* and *N*_*i*,*j*_ is the total number of conformations in the bin (*i*, *j*). Thus, the relative fraction is dimensionless and gives the occupancy proportion of each model in each bin. To avoid border effects, bins with poor statistics were neglected. The result is shown in Figure 4, in which the individual relative fraction is shown through a colormap superimposed on the conformational space points drawn in gray. It is possible to see that most regions of the conformational space are shared among all models, with each model having a relative occupancy of around 0.2. Nevertheless, every model populates regions of the conformational space with high exclusivity. For the A1_A_ model, two large regions are observed that span about 5% of its conformational space and have a relative fraction equal to 1.

**Figure 4:**
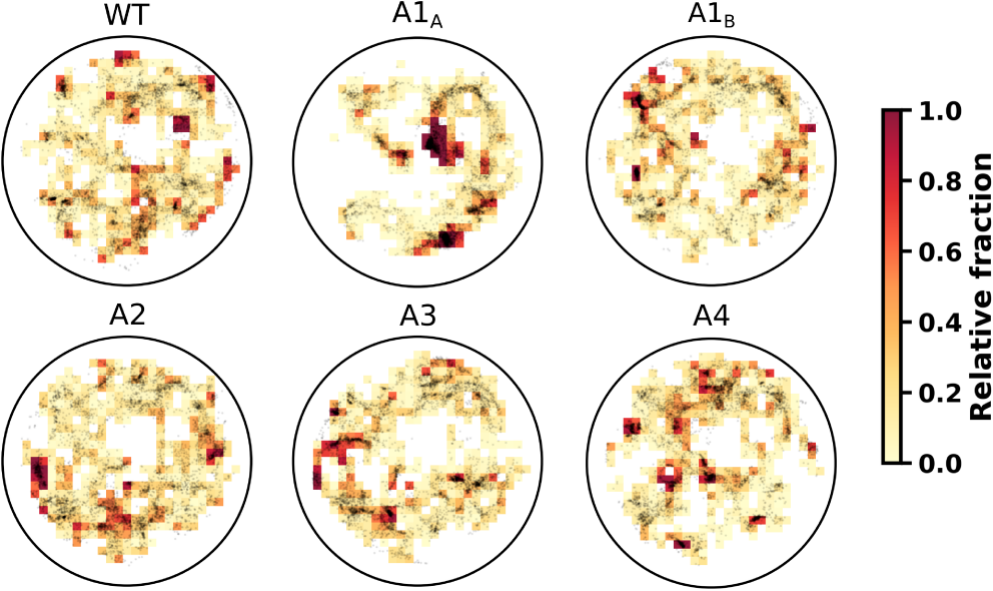
Relative fraction of each H4 tail model across the projected conformational space. The grid is superimposed on the conformational space, with each bin colored according to the local relative fraction of conformations. Yellow bins represent low relative fractions, while dark red bins indicate uniquely populated regions with a relative fraction of 1.

We also estimated how much of the conformational space is shared among the models by defining a density overlap measure. First, we normalized the relative fraction of each model, $f_{i,j}^{w}=F_{i,j}^{w}/\sqrt{\underset{i,j}{\sum }F_{i,j}^{w}\cdot F_{i,j}^{w}}$ , and then we calculated the density overlap between models *w* and *z* by

3 $$O^{w,z}=\underset{i,j}{\sum }f_{i,j}^{w}\cdot f_{i,j}^{z}$$

*O* ^*w*,*z*^ is dimensionless, equals zero if the density distributions are completely independent, and equals one if they are identical. Table 1 summarizes the density overlap between every pair of the studied H4 tail models. The overlap between the WT and A2 models is the largest, as expected, because their individual conformational spaces are the most widely spread across the effective conformational space. The lowest overlap occurs between the A1_A_ and A4 models.

**Table 1:** Density Overlap between the H4 Tail Models

	WT	A1_A_	A1_B_	A2	A3	A4
WT	1.00	0.18 ± 0.03	0.35 ± 0.06	0.47 ± 0.04	0.42 ± 0.05	0.39 ± 0.05
A1_A_	-	1.00	0.13 ± 0.02	0.15 ± 0.02	0.12 ± 0.02	0.11 ± 0.02
A1_B_	-	-	1.00	0.34 ± 0.04	0.33 ± 0.05	0.30 ± 0.05
A2	-	-	-	1.00	0.45 ± 0.05	0.37 ± 0.04
A3	-	-	-	-	1.00	0.38 ± 0.05
A4	-	-	-	-	-	1.00

Finally, we address the question of how much acetylation changes the conformational space heterogeneity of the studied models. To account for this, we calculated projection entropies, considering the point distributions over the bins. First, for each model (*w*), we defined the probability distribution of having $n_{i,j}^{w}$ conformations in the bin (*i*, *j*) as $p_{i,j}^{w}=n_{i,j}^{w}/N^{w}$ , where *N* ^*w*^ is the total number of conformations of the model *w*. The projection entropy is then given by

4 $$H^{w}=-\underset{i,j}{\sum }p_{i,j}^{w}\ln p_{i,j}^{w}$$

This entropy is also dimensionless. For the chosen grid size, it would reach a maximum value of *H*_max_ = 6.59 if conformations were uniformly distributed across the bins. To compare all of the models, we also normalized the entropy values by dividing them by *H*_max_.

The force-scheme technique^56^ employed in ELViM is not deterministic, rendering slightly different projections for different runs. These differences, nevertheless, do not affect global properties of the projection^34,37^ and are considered to be projection noise. Nevertheless, to account for these noise effects and make sure that projection heterogeneity is in fact changed by acetylation, we ran 50 independent ELViM projections (not shown) and calculated the projection entropy for each projection (Figure S9). The mean normalized entropy and the standard deviation of each projection are shown in Figure 5. It should be noted that the low standard deviation corroborates the robustness of the projections. The WT has the highest mean entropy, while the monoacetylated A1_A_ has the lowest one, as it underwent the greatest reduction in its accessible space. The monoacetylated A1_B_ also has an entropy lower than that of the WT. Comparing the A2, A3, and A4 models, we see that increasing the number of acetylations slightly lowers the entropy of the distribution, as previously discussed.

**Figure 5:**
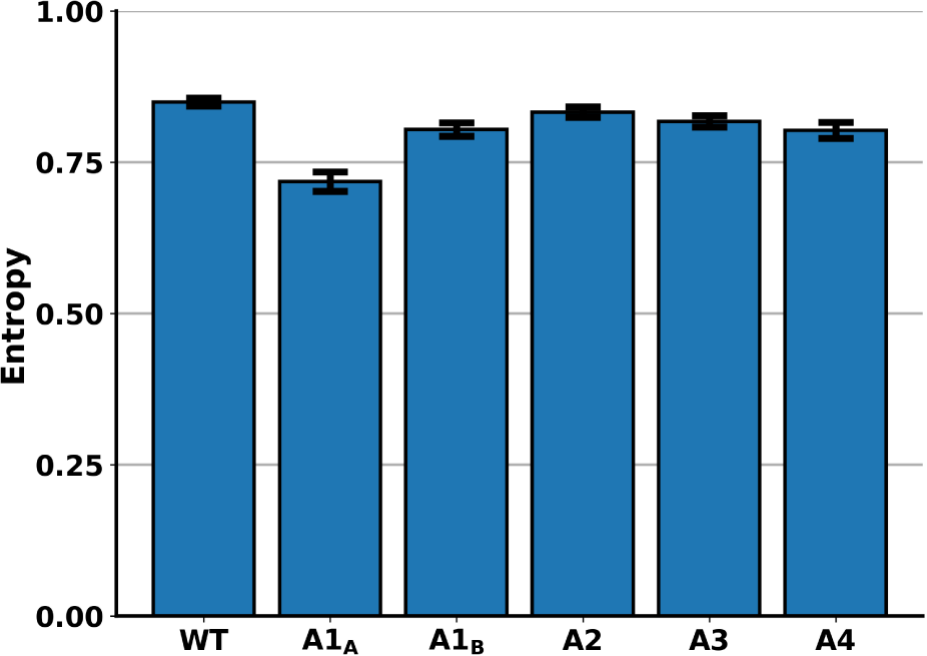
Normalized projection entropy of the H4 models. Mean normalized projection entropy for each H4 tail model, with standard deviation from 50 independent projections. Entropy values were calculated using eq 4 and normalized by the maximum entropy *H*_max_ = 6.59, which corresponds to a uniform distribution across the grid.

We should note that the presented results may be sensitive to the choice of the dimensionality reduction method, which can prioritize either local or global aspects of the high-dimensional conformational landscape. Additionally, different featurization methods, such as using Cartesian coordinates or dihedral angles, or employing distinct distance metrics, can significantly influence the resulting representation.^59,60^ Comparing these methods is a challenge, as existing quality metrics^61^ primarily assess algorithm efficiency rather than how well biomolecular topography is represented.^60^ PCA,^62,63^ a well-known and widely used technique, aims to capture the most significant variance in the data and identify collective motions, but it may fail to represent nonlinear properties.^64,65^ Nonlinear methods attempt to address this limitation by better preserving complex relationships within the data.^64,65^ For instance, t-SNE^66^ has been shown to perform well in clustering the heterogeneous ensembles of IDPs.^67^ However, the method mainly focuses on preserving the local neighborhood structure of the high-dimensional space and may not capture the global organization of the landscape.^67^

To illustrate these differences, Figure S10 presents a comparison of the single effective space of H4 tails as generated by PCA, dPCA,^68^ and t-SNE. The subspaces populated by each individual model are compared in Figure S11. Finally, we selected the region with the highest relative fraction of each model to obtain an LCS, which is shown in all projections in Figure S12. Although structurally diverse, LCSs A2 and A1_B_ are projected as superimposed by PCA, while the LCSs for WT and A3 are superimposed in dPCA space. All selected LCSs (Figure S12) are also identified as separate clusters by t-SNE. However, while t-SNE may better identify clusters, it does not preserve the global structure of the landscape. ELViM, in contrast, does not aim at clustering but offers a good balance between local and global aspects, ensuring that reaction coordinates, such as the radius of gyration and the fraction of native contacts, change smoothly across the projection while maintaining a continuous organization of conformations. This facilitates ensemble comparisons and the identification of exclusively populated regions.

### Impact of Nucleosome Binding on H1 Histone Conformation Landscape

In this section, we applied ELViM to explore the effective conformational space of the full-length histone H1 bound to the entire nucleosomal particle. The α-carbon coordinates of histone H1 were extracted and subsampled, as described in the methodology, for subsequent ELViM analysis. The effective conformational space generated by ELViM is shown in Figure 6A, with points representing each conformation colored according to the *R*_g_ values. Although the high flexibility of the H1 tails is reflected in great variation in *R*_g_ values, ELViM projects a conformational space in which *R*_g_ values vary smoothly across the space. To illustrate this conformational variability across the effective conformational space, we show in Figure 6B five LCSs selected from arbitrarily high-density regions. Here, each LCS is composed of 20 conformations superimposed as described in the Methods section. The conformations selected for each LCS are colored red in the projection, while the corresponding backbones are colored red, with the N-terminus also in red.

**Figure 6:**
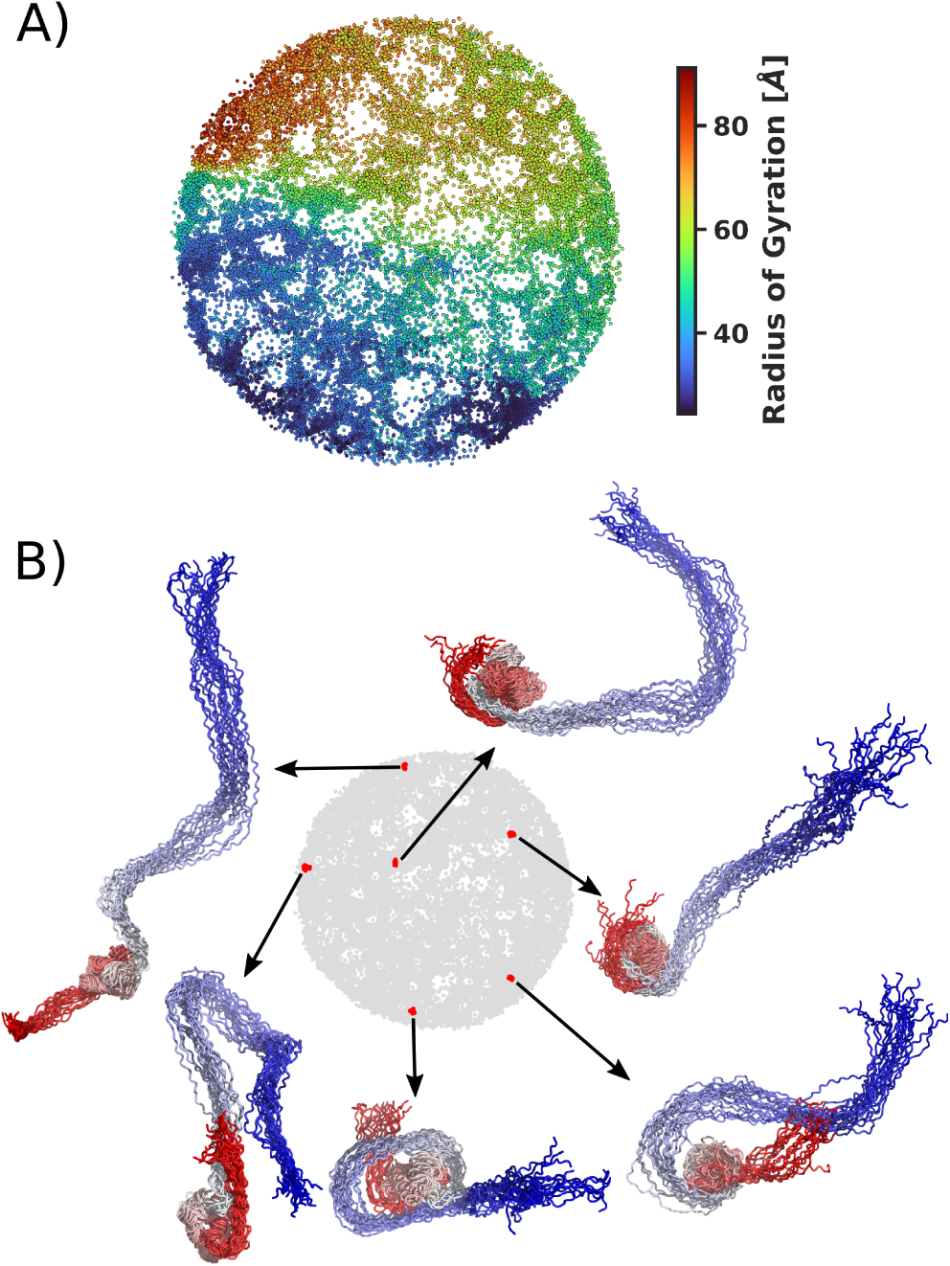
Effective conformational space for full-length H1. (A) Each sampled conformation is represented by a point in the projected space, with points colored based on *R*_g_ values. (B) Local conformational signatures. Each LCS displays 20 aligned conformations, with points colored in red to highlight their location in the projection. The backbones are depicted, and the N-terminal residues are drawn in red.

Based on the energy landscape projection, we explored how the binding mode to the nucleosome particle influences the H1 structural heterogeneity. To achieve this, we divided the space surrounding the nucleosome into eight cylindrical sectors, as depicted in Figure 7A. For each simulation frame, the H1 position was defined according to the cylindrical coordinates of the center of mass of the H1 globular domain relative to the center of mass of the nucleosome core particle. Sector 0 is assigned to unbound states, while in sector 1, H1 binds near the dyad. In sector 2, H1 escapes from the dyad and is close to the acidic patch on the histone core. Other sectors account for frames where H1 is near or has drifted away from the nucleosome. Sector occupancy frequency, considering all simulated frames, is sector 0: 10.1%, sector 1: 78.3%, sector 2: 9.8%, sector 3: 1.3%, and sector 7: 0.5%. Sectors 4, 5, and 6 were not visited during the simulation.

**Figure 7:**
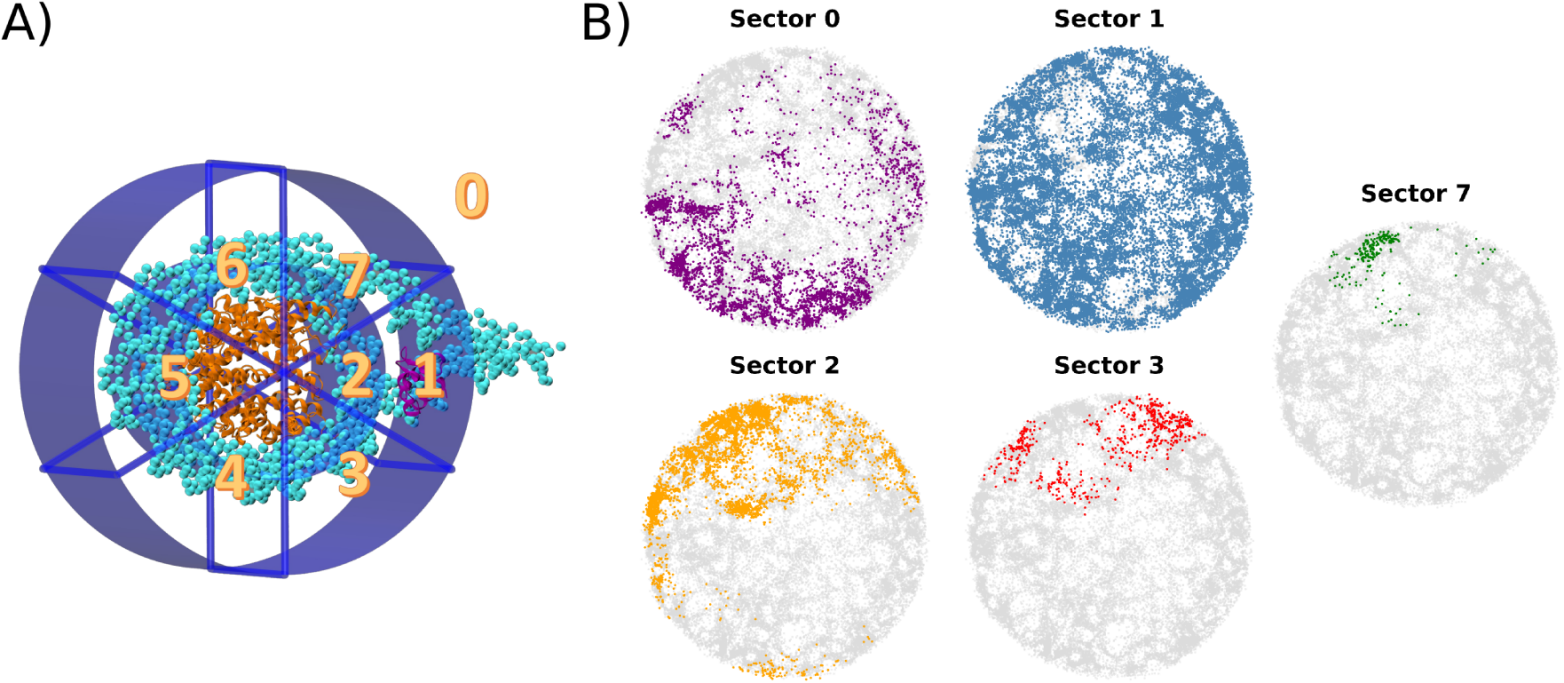
Nucleosome binding sectors. (A) The space surrounding the nucleosome was divided into eight cylindrical sectors. (B) Conformations sampled from each sector are displayed in distinct colors over the entire conformational space, shown in gray. Sectors not illustrated in this figure indicate those that were not visited during the simulation. Sector occupancy frequencies, considering all simulated frames, are sector 0: 10.1%, sector 1: 78.3%, sector 2: 9.8%, sector 3: 1.3%, and sector 7: 0.5%.

The impact of the disordered domains of H1 on regulating chromatosome structure has been extensively discussed by Hao et al.^44^ Here, we explore whether different binding modes can give rise to distinct conformational preferences, despite H1 remaining highly disordered even in the bound state.

In Figure 7B, we use different colors to highlight the sectors from which each conformation was sampled. Given that most conformations were sampled from sector 1, it is expected that the effective conformational space is primarily occupied by this sector. Interestingly, however, conformations are distributed throughout the entire space, contrasting with the uniquely populated regions observed in the previously discussed H4 models. The remaining sectors are restricted to smaller neighborhoods within the conformational space. This restriction in conformational heterogeneity may result from steric effects, binding-induced conformational changes, or limited sampling within these sectors.

Comparing sectors 0, 1, and 2, it is noteworthy that regions of higher density are located in different areas of the conformational space, potentially indicating conformational preferences associated with specific binding modes. A detailed analysis of these conformational preferences is provided in Figures S13 and S15, where we present the LCS and contact frequency for the highest-density regions within each sector.

## Conclusion

In this study, we applied ELViM to analyze the conformational dynamics of intrinsically disordered histone H4 tails and the linker histone H1. Our results highlight the effects of acetylation and nucleosome binding on the conformational landscape of H4 tails and the linker H1, respectively.

The ELViM projection of the H4 tail revealed significant variations in the accessible conformational space between the wild type and various acetylated forms. Notably, acetylation tends to reduce the overall conformational heterogeneity, as evidenced by the restricted conformational spaces observed for acetylated models compared with the wild type. This reduction in heterogeneity is consistent with previous findings that cumulative acetylation reshapes the energy landscape of IDPs. Furthermore, certain acetylated forms, such as the monoacetylated A1_A_, were found to populate unique regions of the conformational space, suggesting that specific acetylation patterns can induce distinct conformational preferences and potentially unique biological functions.

The ELViM analysis for linker histone H1, simulated in complex with the nucleosome particle, indicates that the binding mode can lead to changes in conformational preferences, reshaping the local minima within the conformational landscape. In this case, binding to different sectors around the nucleosome restricts H1’s conformational variability, highlighting the role of binding in defining the functional states of intrinsically disordered proteins (IDPs).

Overall, our study underscores the utility of ELViM in dissecting the complex conformational landscapes of IDPs and illustrates the intricate interplay between post-translational modifications and protein dynamics. These insights not only enhance our understanding of histone biology but also pave the way for future investigations into the functional implications of IDP conformational plasticity and its regulation by cellular modifications.

## Supplementary Material



## Data Availability

ELViM is available on GitHub (https://github.com/VLeiteGroup/ELViM). The following free software were used: VMD^69,70^ and Pymol^71^ for structural visualization, and MDTraj^72^ for handling MD trajectories. The MD trajectory data used in the analysis is available upon request.

## Notes

H4 and H1 systems, such as preferential ensembles and contact maps; for the H4 models, it also features an analysis of the acidic patch SASA, helical content, and a plot of projection entropy for each ELViM run; additionally, a comparison of the H4 systems with other established techniques, namely PCA, dPCA, and t-SNE (PDF)
