# Supplementary material for: Energy Landscapes and Structural Plasticity of Intrinsically Disordered Histones
Source: J Chem Inf Model. 2025 Aug 6;65(16):8679–87. doi: 10.1021/acs.jcim.4c02269 (PMC12381848; doi:10.1021/acs.jcim.4c02269)
Supplement: Supplementary file 1 [file ci4c02269_si_001.pdf]

Supporting Information - Energy Landscapes and Structural Plasticity of Intrinsically Disordered Histones

Rafael G. Viegas,[†,‡] Hao Wu,[¶] Murilo N. Sanches,[‡] Garegin A. Papoian,[§,‖] and Vitor B.P. Leite[*,‡]

†*Federal Institute of Education, Science and Technology of São Paulo (IFSP), Catanduva, SP, 15.808-305, Brazil*

‡*Department of Physics, São Paulo State University (UNESP), Institute of Biosciences, Humanities and Exact Sciences, São José do Rio Preto, SP, 15054-000, Brazil*

¶*Oncology Chemistry, AstraZeneca, 35 Gatehouse Dr, Waltham, MA 02451, USA*

§*Biophysics Program, Institute for Physical Science and Technology, University of Maryland, College Park, Maryland 20742, United States*

‖*Department of Chemistry and Biochemistry, University of Maryland, College Park, Maryland 20742, United States*

E-mail: vitor.leite@unesp.br

Supporting Figures

In this section, we present the Local Conformational Signatures (LCSs) for each H4 tail model. A detailed definition of the LCS can be found in the methodology section of the main manuscript. Each figure replots the effective phase space of each model, with points colored according to local density, calculated using Gaussian Kernel Density Estimation (KDE).

Regions of highest density were identified, and for each region, we extracted LCSs consisting of 20 conformations. Additionally, we provide average contact maps, or contact frequencies, for each LCS. Contacts were defined between Cα atom pairs using a distance cutoff of 8 Å for the H4 model. The same analysis was performed to highlight the conformational preferences of each binding mode of the full H1 linker histone, with contacts defined by a cutoff distance of 10 Å for the H1 model.

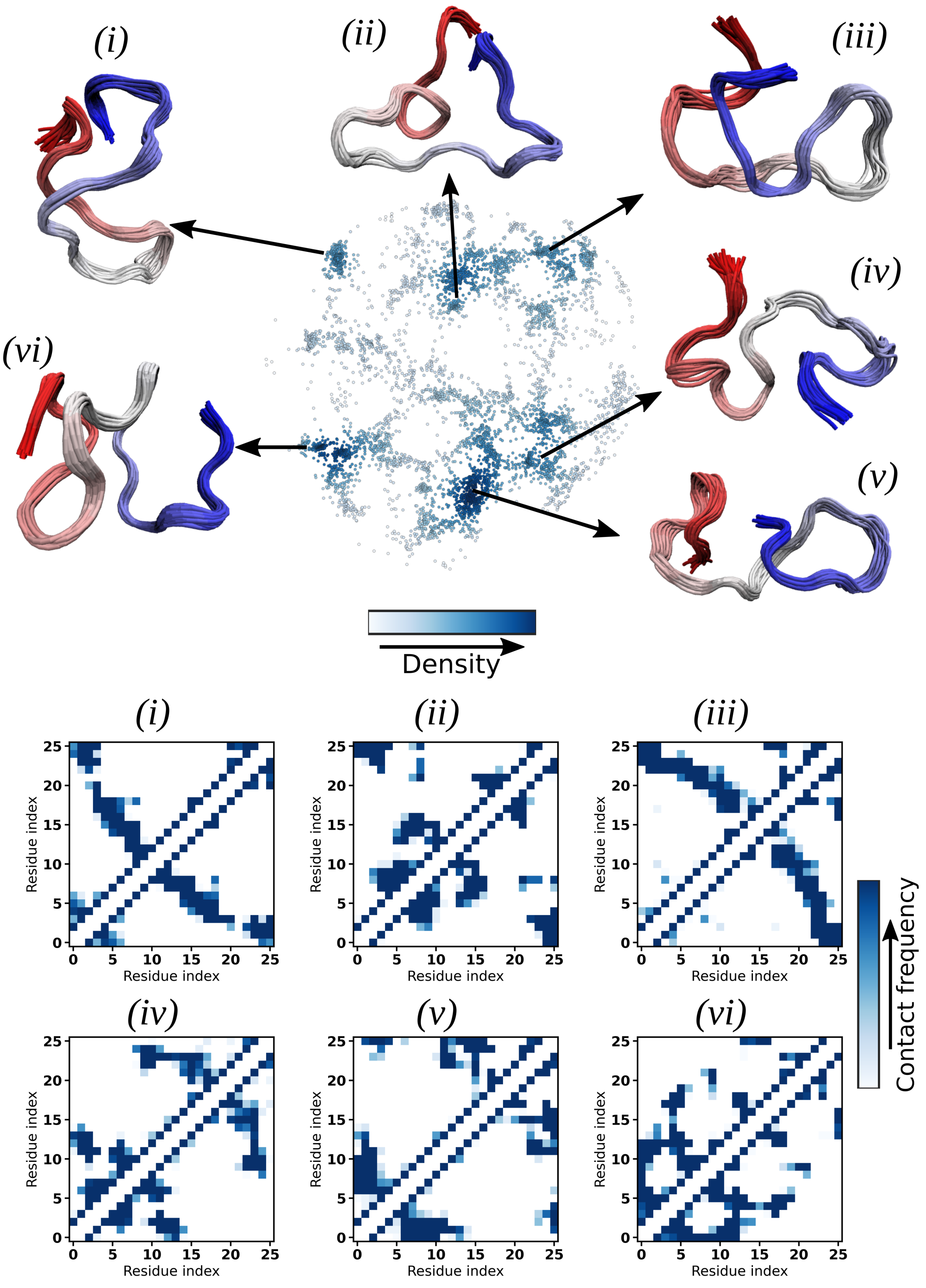

Figure S1: Conformational signatures and contact frequency for the wild-type form of the H4 histone tail.

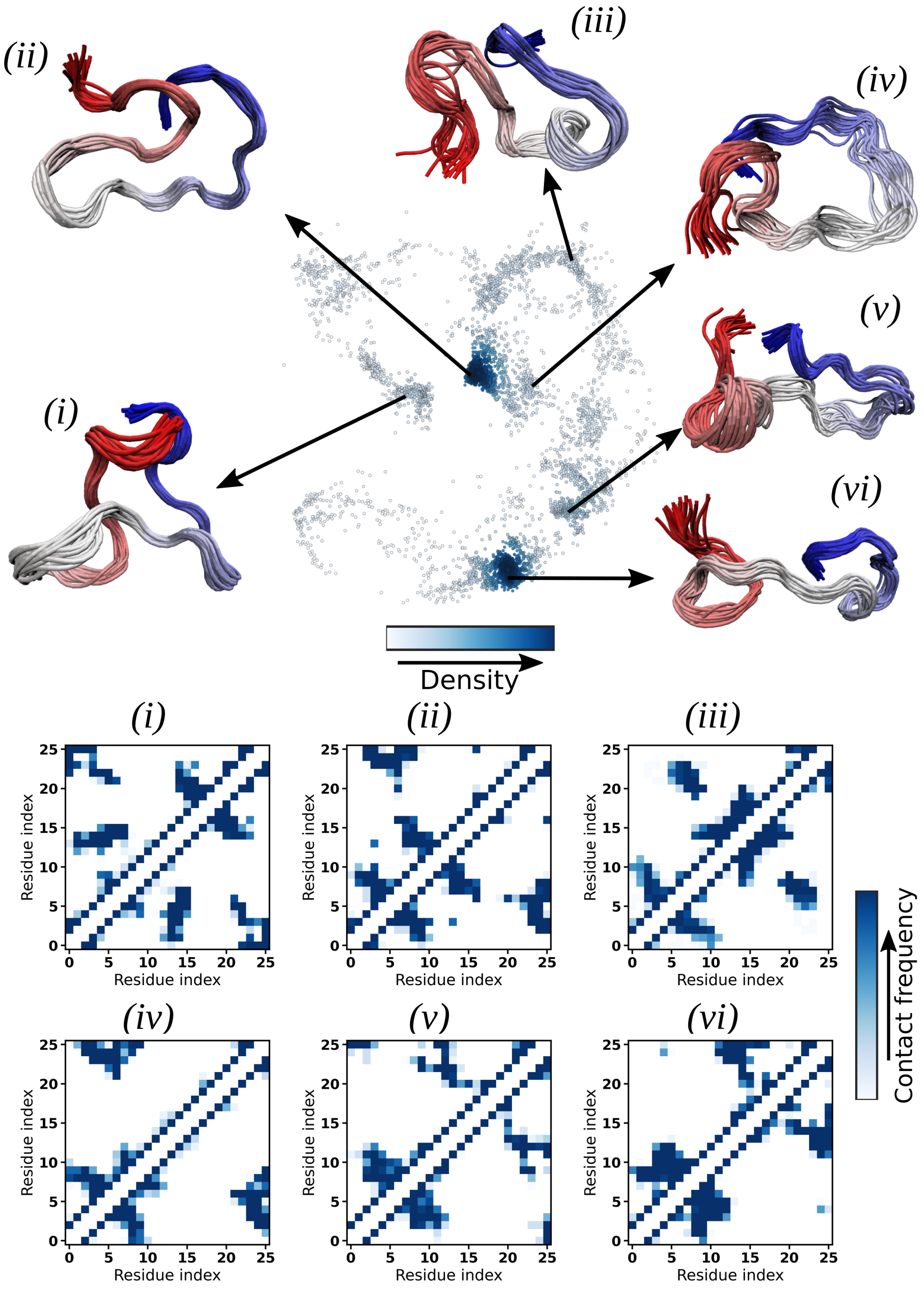

Figure S2: Conformational signatures and contact frequency for the acetylated $A1_A$ form of the H4 histone tail.

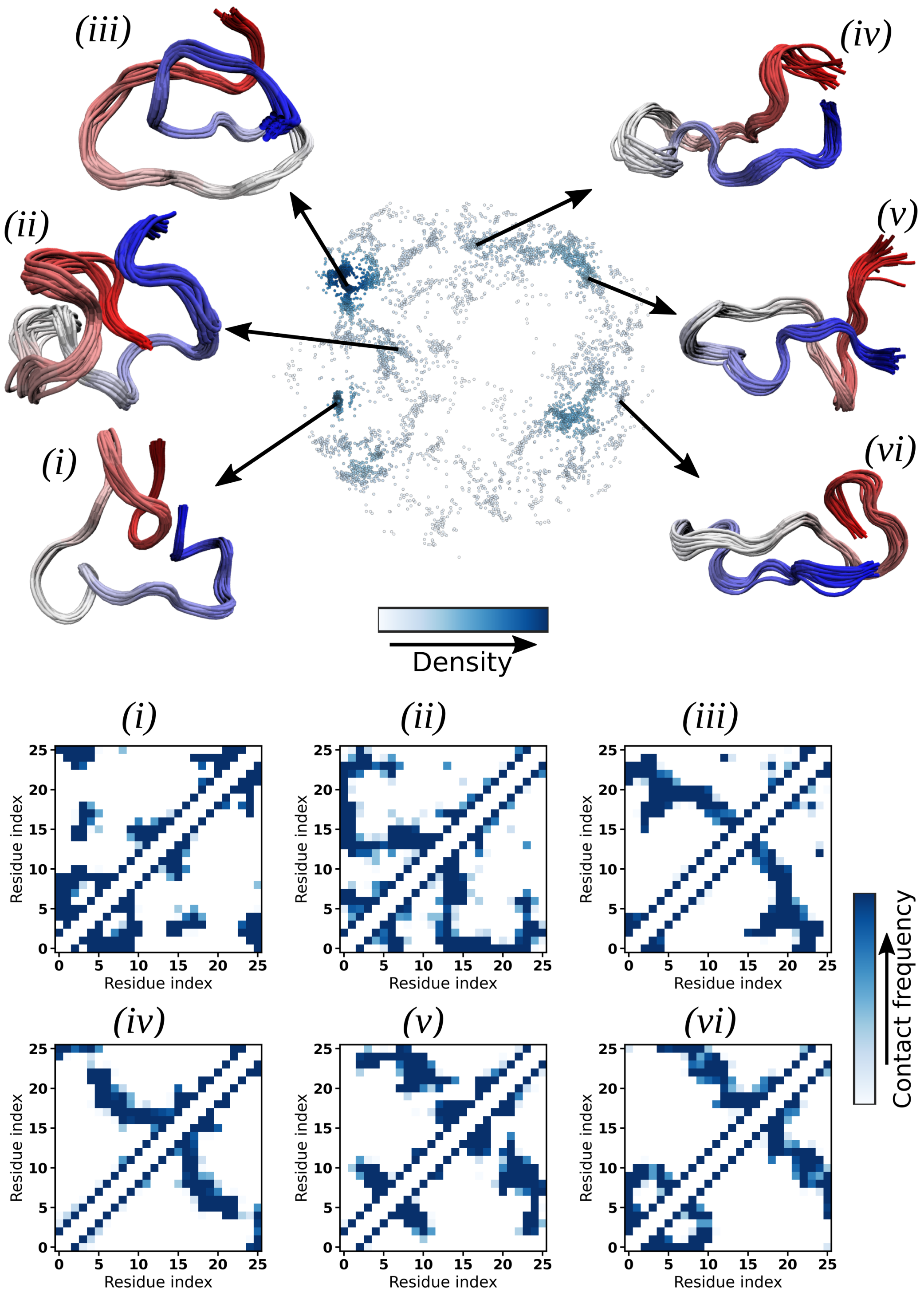

Figure S3: Conformational signatures and contact frequency for the acetylated $A1_B$ form of the H4 histone tail.

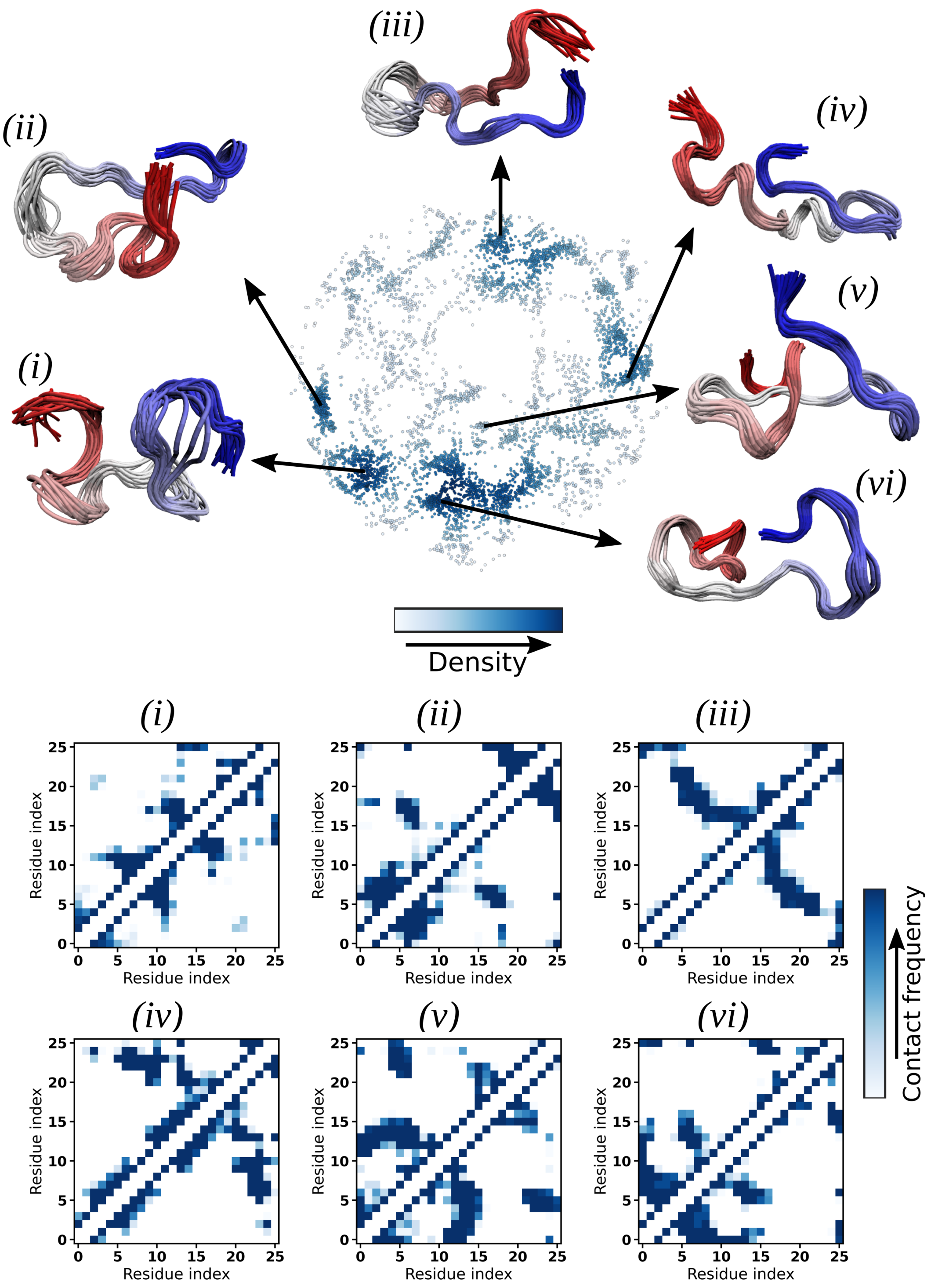

Figure S4: Conformational signatures and contact frequency for the acetylated A2 form of the H4 histone tail.

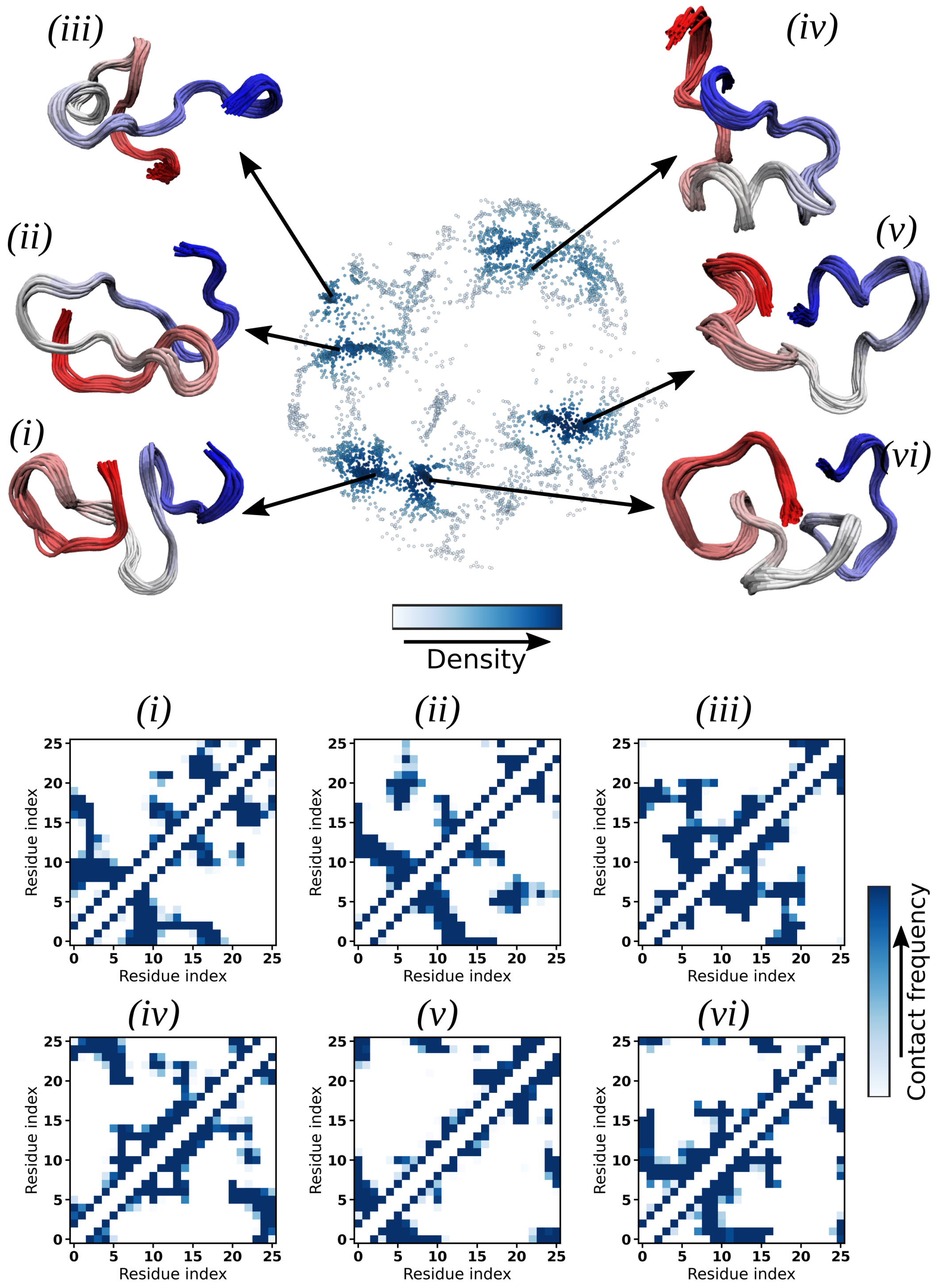

Figure S5: Conformational signatures and contact frequency for the acetylated A3 form of the H4 histone tail.

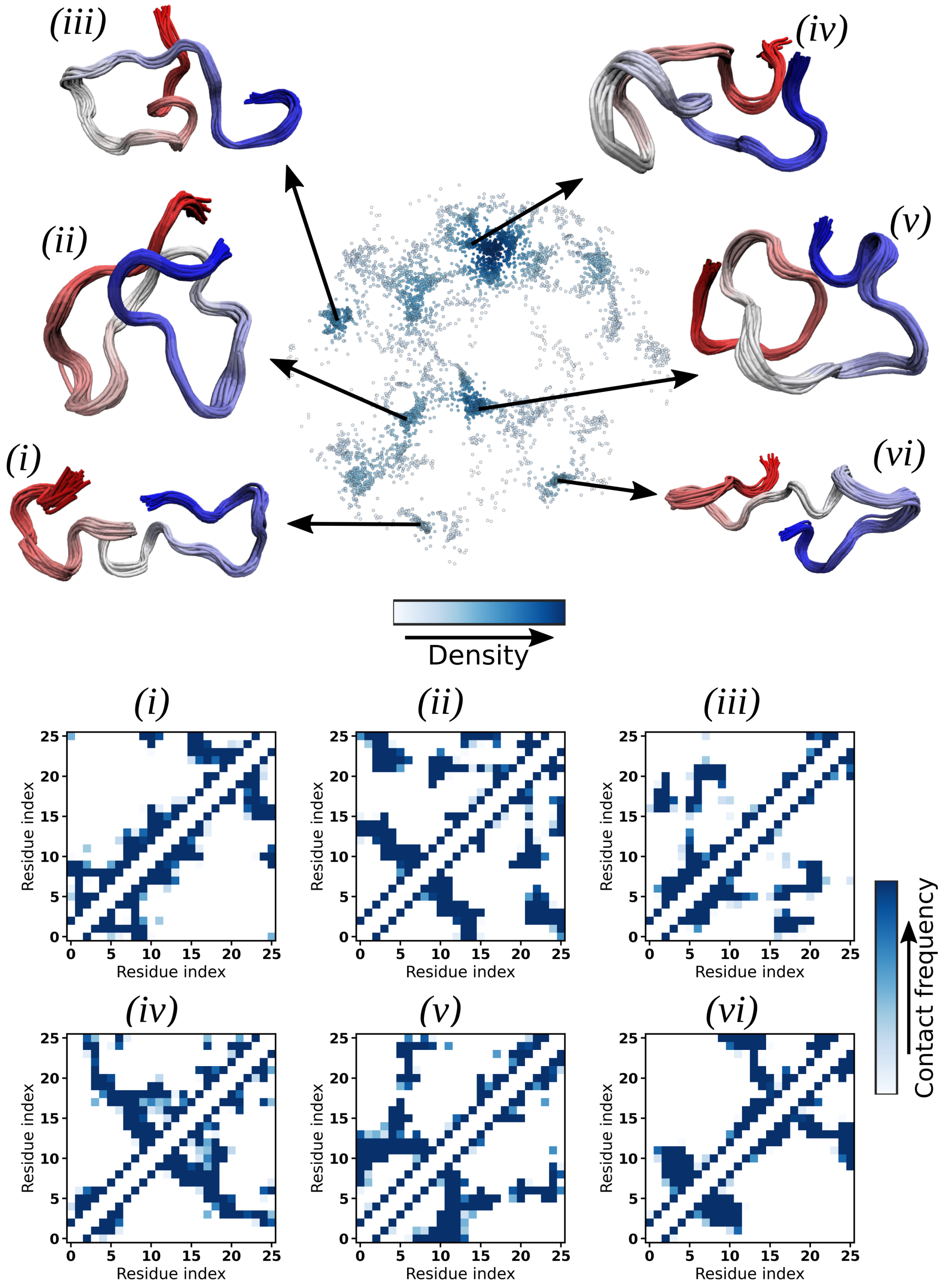

Figure S6: Conformational signatures and contact frequency for the acetylated A4 form of the H4 histone tail.

/

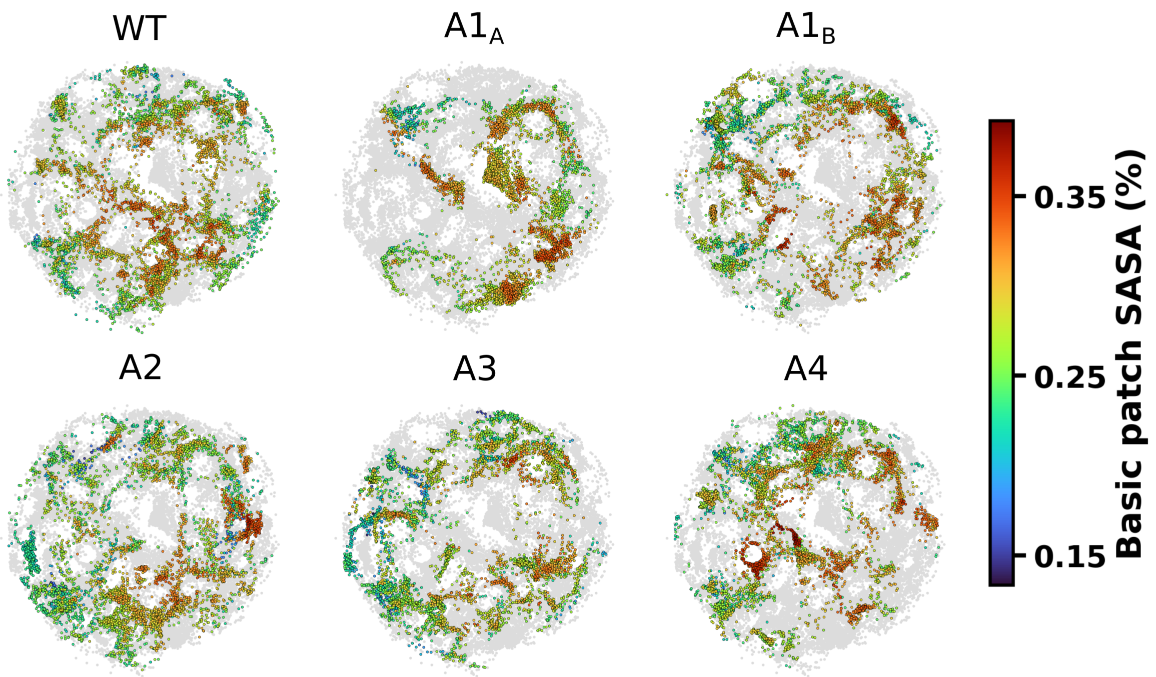

Figure S7: Basic Patch Accessibility in H4 Histone Tail Models. The solvent-accessible surface area (SASA) of the basic patch (residues 16–20, KRHRK) is presented as a percentage of the total SASA for each conformation. This basic patch is a positively charged region which can interact with acidic patches on both DNA and H2A/H2B. The SASA was estimated using the Shrake–Rupley algorithm,[1] as implemented in MDTraj.[2]

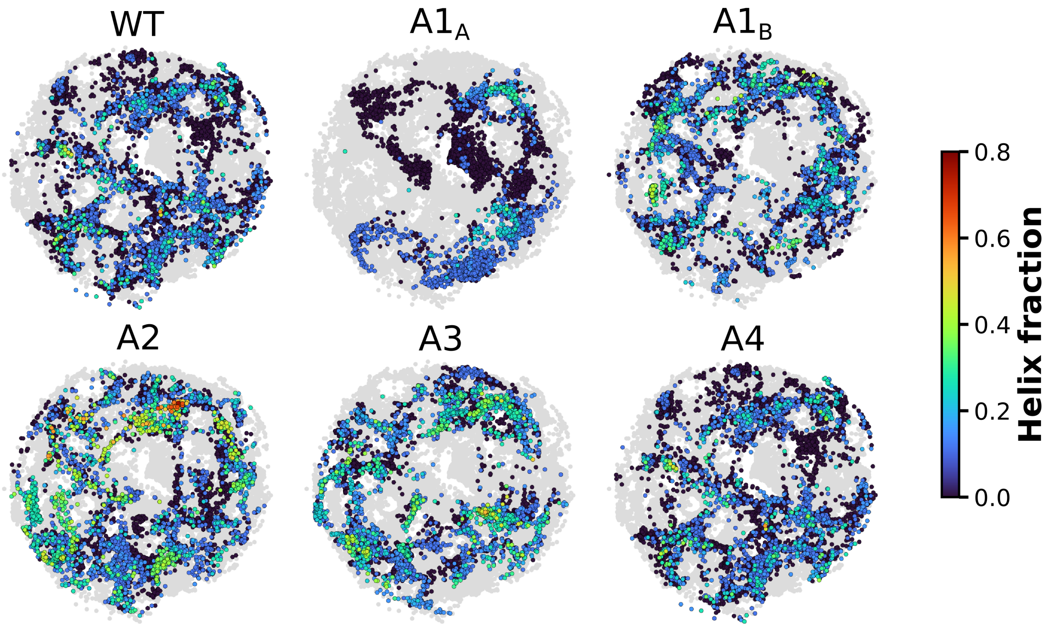

Figure S8: Helix Fraction for H4 Histone Tail Models. The secondary structure content was estimated using DSSP,[3] as implemented in MDTraj.[2] The helical content includes both α-helices and 3_{10}-helices. A per-residue analysis is available in reference.[4]

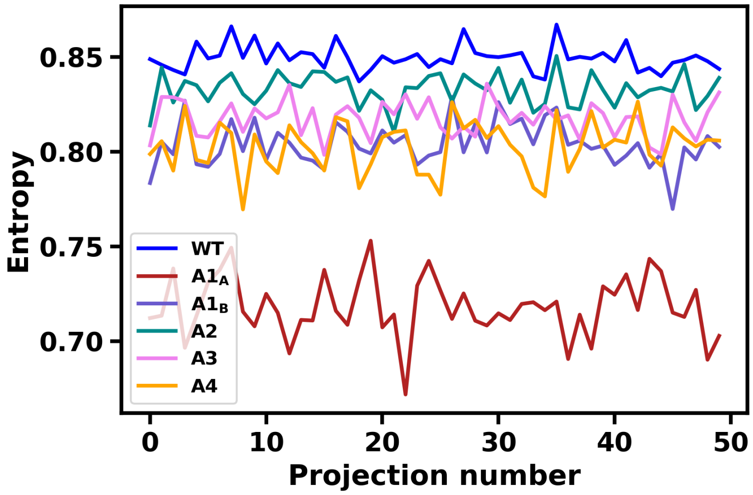

Figure S9: H4 Histone Tails: Projection Entropy. The projection entropy for each model was estimated from 50 independent projection runs. For a detailed definition, as well as the mean and standard deviation values, please refer to the main manuscript.

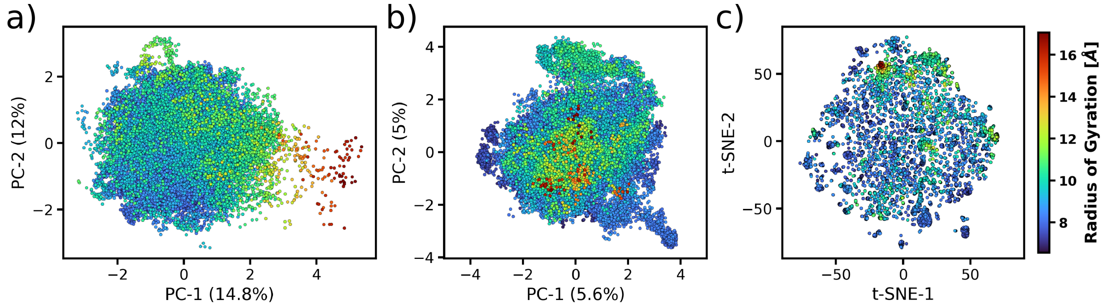

Figure S10: H4 Histone Tails: Reduced spaces produced by other dimensionality reduction methods. (a) PCA of the Cα Cartesian coordinates. To remove translation and rotation, conformations were centered on the MD trajectory average structure, and this process was refined until convergence was reached. (b) PCA based on dihedral angles Φ and Ψ. To account for angle periodicity, dihedrals were mapped onto the unit circle by calculating the sine and cosine of each dihedral angle. Axis labels indicate the explained variance of each component. Both PCA analyses were performed using the Scikit-learn[5] library. (c) t-SNE was performed following the protocol described in ref.[6] The input distance matrix was computed as the RMSD of Cα carbons. Analysis of the silhouette coefficient resulted in a perplexity of 400 for t-SNE and K=100 for the K-means algorithm. Other parameters were kept as described in ref.[6] To facilitate comparison, points were colored based on the R_g values, as in the ELViM projection.

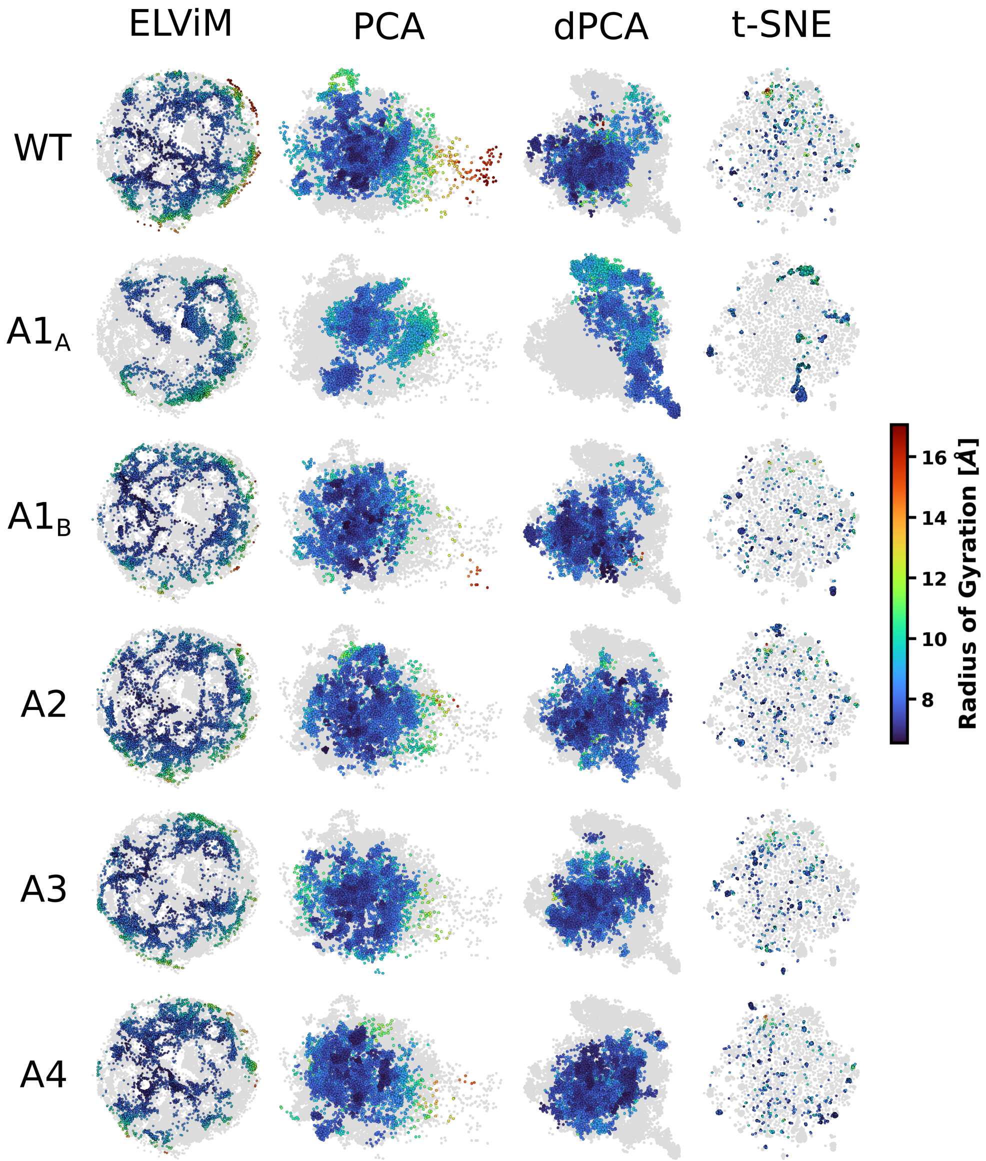

Figure S11: H4 Histone Tails: Effective phase space of each H4 model using different dimensionality reduction methods. To aid visual comparison, all axes have been omitted. Dots representing each tail model are colored by R_g values, with the global space shown in gray. For details regarding PCA and t-SNE, see the previous figure.

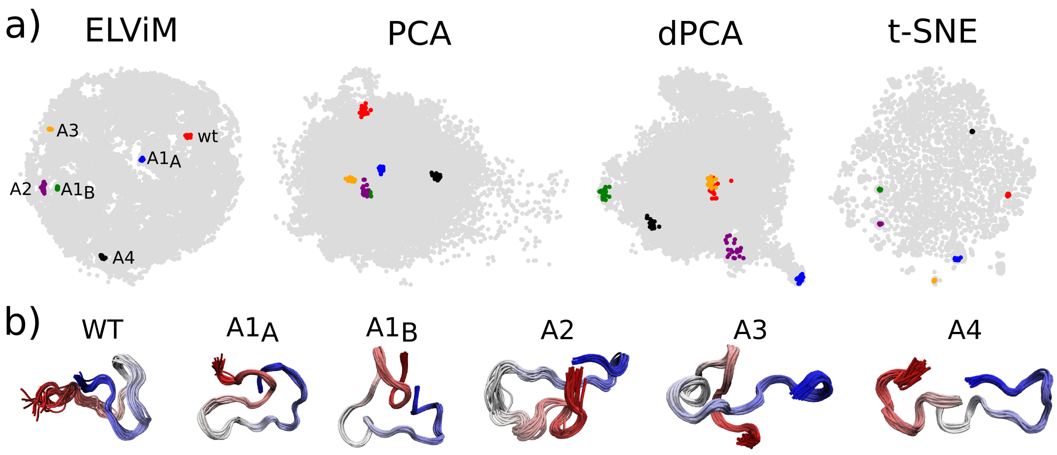

Figure S12: H4 Histone Tails: Relative position of the conformational signature with the highest Relative Fraction. An LCS was selected from the region with the highest Relative Fraction (Figure 4 of the manuscript). The dots representing each conformation are shown in (a), while the superposed conformations are presented in (b).

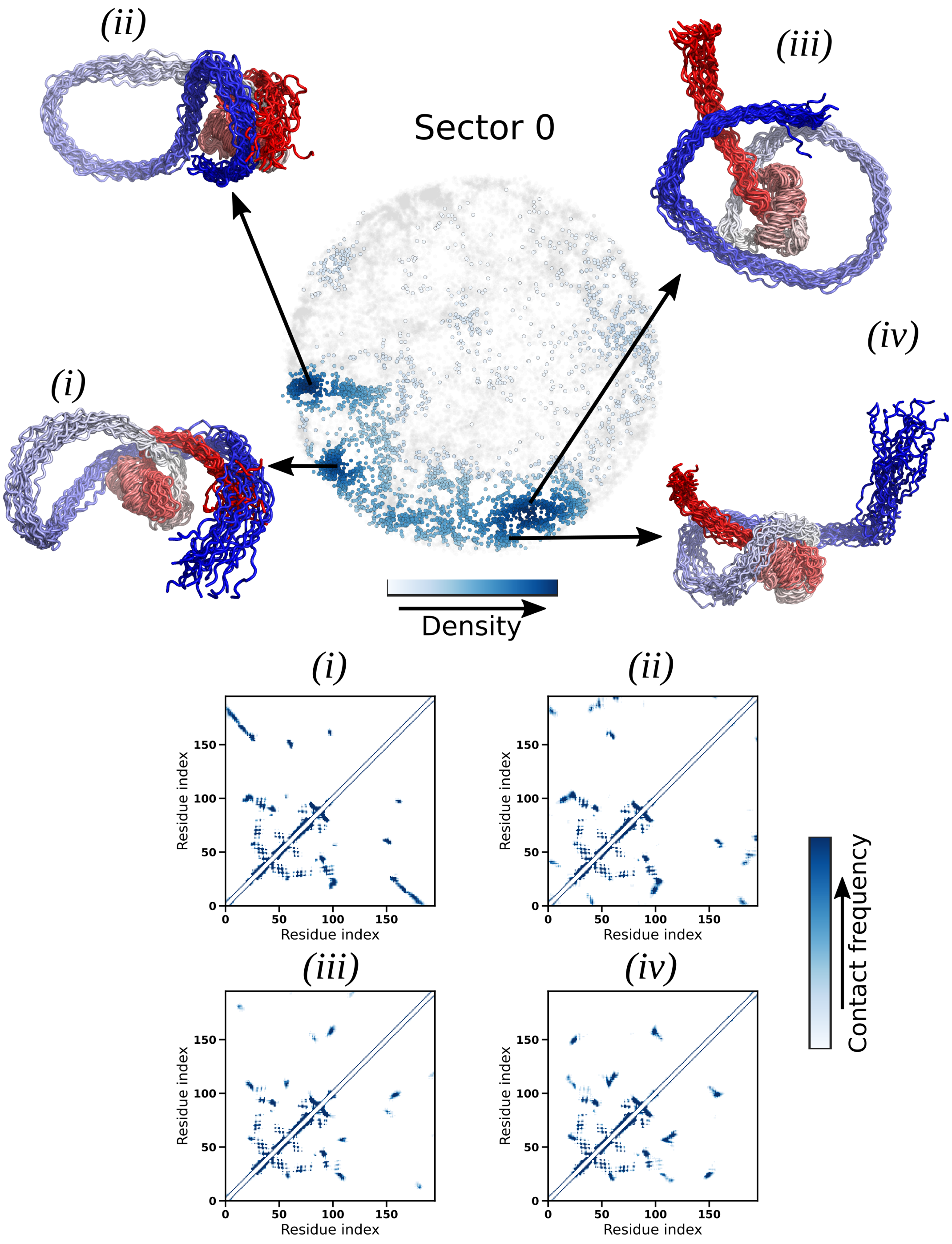

Figure S13: Conformational signatures and contact frequency for sector 0 in the H1 effective phase space.

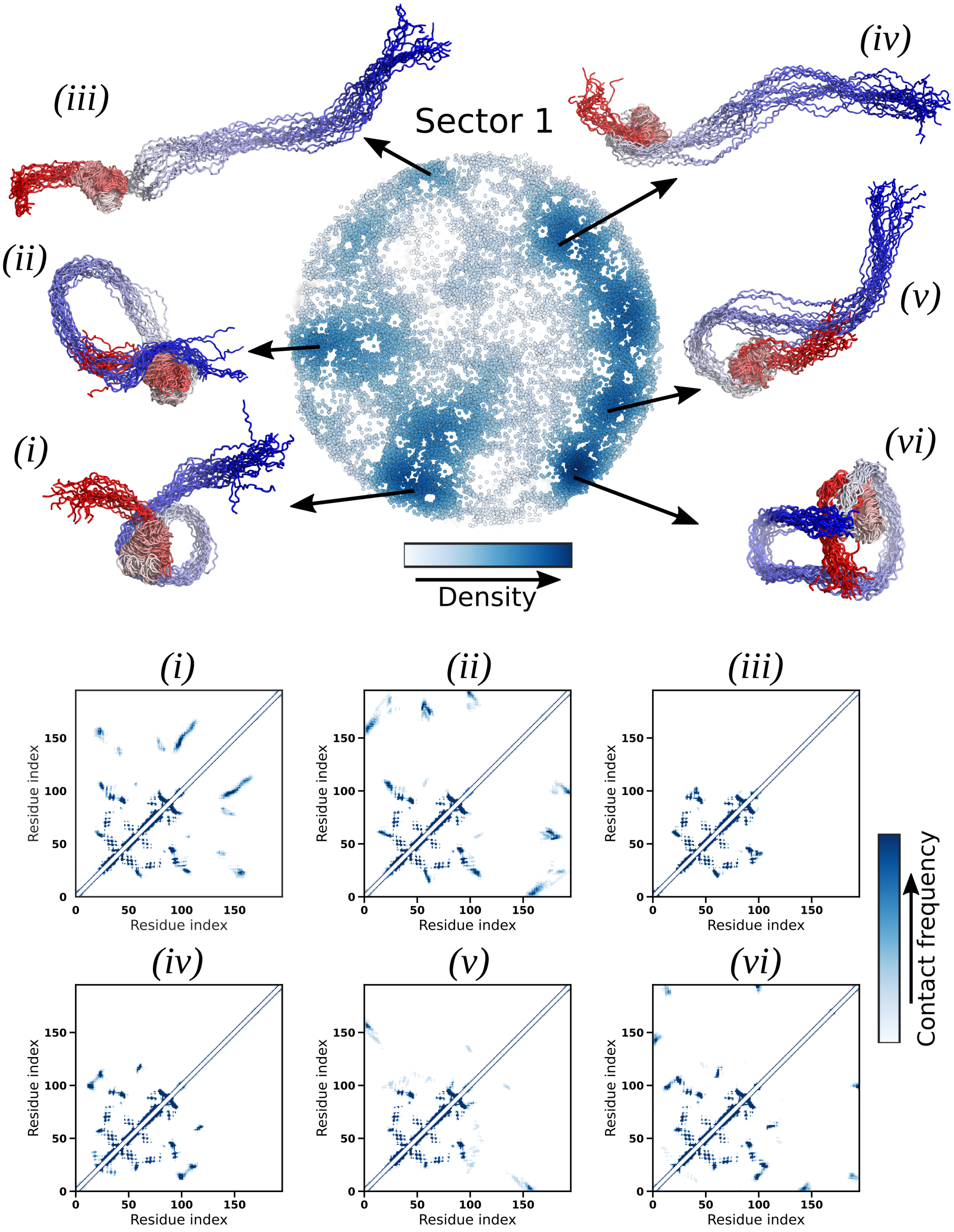

Figure S14: Conformational signatures and contact frequency for sector 1 in the H1 effective phase space.

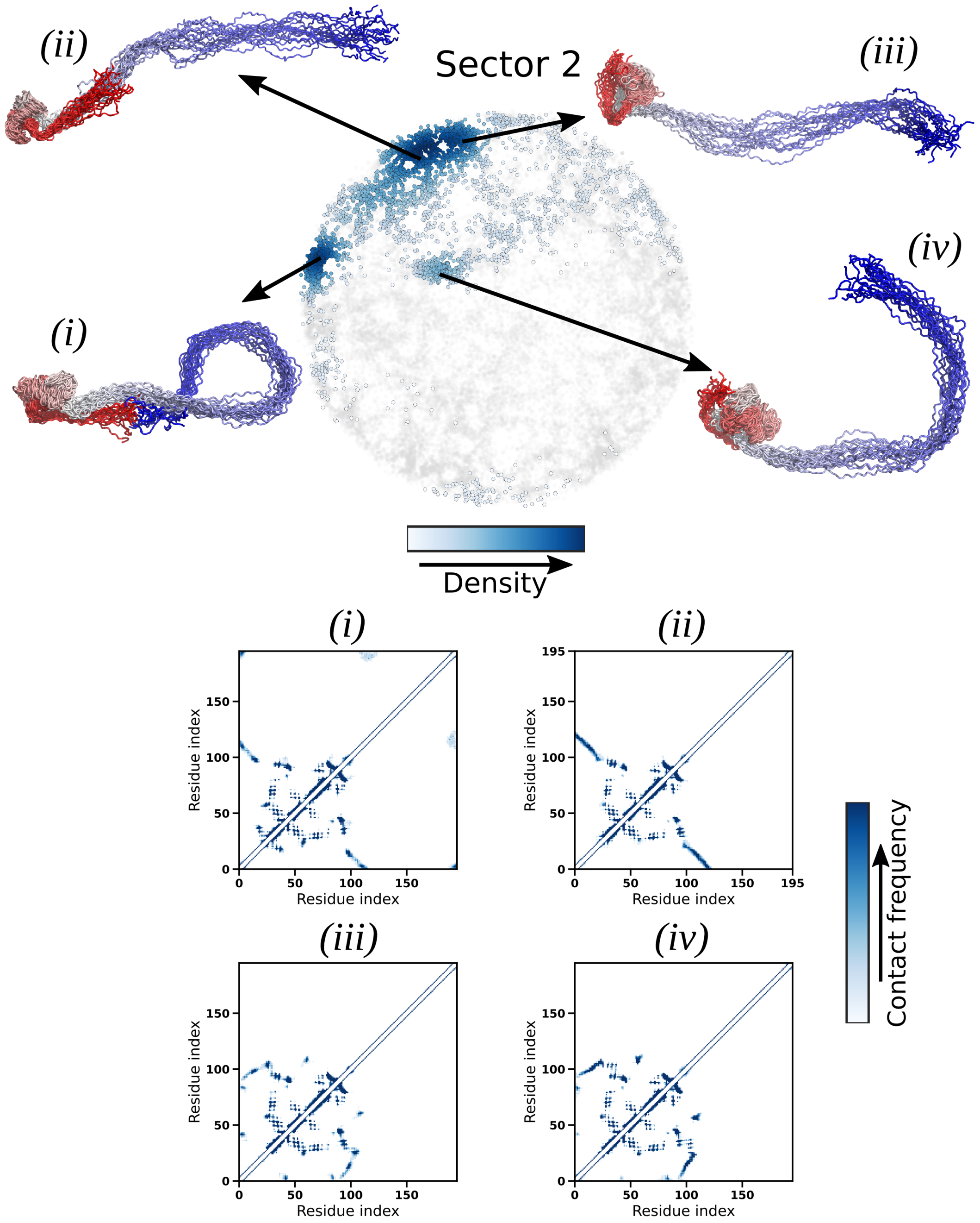

Figure S15: Conformational signatures and contact frequency for sector 2 in the H1 effective phase space.

References

(1) Shrake, A.; Rupley, J. Environment and exposure to solvent of protein atoms. Lysozyme and insulin. *J. Mol. Biol.* **1973**, *79*, 351–371.

(2) McGibbon, R. T.; Beauchamp, K. A.; Harrigan, M. P.; Klein, C.; Swails, J. M.; Hernández, C. X.; Schwantes, C. R.; Wang, L.-P.; Lane, T. J.; Pande, V. S. MDTraj: A Modern Open Library for the Analysis of Molecular Dynamics Trajectories. *Biophys. J.* **2015**, *109*, 1528 – 1532.

(3) Kabsch, W.; Sander, C. Dictionary of protein secondary structure: Pattern recognition of hydrogen-bonded and geometrical features. *Biopolymers* **1983**, *22*, 2577–2637.

(4) Winogradoff, D.; Echeverria, I.; Potoyan, D. A.; Papoian, G. A. The Acetylation Landscape of the H4 Histone Tail: Disentangling the Interplay between the Specific and Cumulative Effects. *J. Am. Chem. Soc.* **2015**, *137*, 6245–6253.

(5) Pedregosa, F. et al. Scikit-learn: Machine Learning in Python. *J. Mach. Learn. Res.* **2011**, *12*, 2825–2830.

(6) Appadurai, R.; Koneru, J. K.; Bonomi, M.; Robustelli, P.; Srivastava, A. Clustering Heterogeneous Conformational Ensembles of Intrinsically Disordered Proteins with t-Distributed Stochastic Neighbor Embedding. *J. Chem. Theory Comput.* **2023**, *19*, 4711–4727.
